# Knowledge exchange in the Pacific: The TROPIC (Translational Research into Obesity Prevention Policies for Communities) project

**DOI:** 10.1186/1471-2458-12-552

**Published:** 2012-07-25

**Authors:** Helen Mavoa, Gade Waqa, Marj Moodie, Peter Kremer, Marita McCabe, Wendy Snowdon, Boyd Swinburn

**Affiliations:** 1 WHO Collaborating Centre for Obesity Prevention, Deakin University, Melbourne, Australia; 2 Fiji School of Medicine, College of Medicine Nursing and Health Sciences, Fiji National University, Suva, Fiji; 3 Deakin Health Economics, Deakin University, Melbourne, Australia; 4 McCaughey Centre, The University of Melbourne, Melbourne, Australia; 5 School of Psychology, Deakin University, Melbourne, Australia; 6 School of Population Health, University of Auckland, Auckland, New Zealand; 7 WHO Collaborating Centre for Obesity Prevention, Deakin University, 221 Burwood Highway, Melbourne, Vic, 3000, Australia

**Keywords:** Policy, Obesity, Knowledge exchange, Knowledge broker, Pacific

## Abstract

**Background:**

Policies targeting obesogenic environments and behaviours are critical to counter rising obesity rates and lifestyle-related non-communicable diseases (NCDs). Policies are likely to be most effective and enduring when they are based on the best available evidence. Evidence-informed policy making is especially challenging in countries with limited resources. The Pacific TROPIC (Translational Research for Obesity Prevention in Communities) project aims to implement and evaluate a tailored knowledge-brokering approach to evidence-informed policy making to address obesity in Fiji, a Pacific nation challenged by increasingly high rates of obesity and concomitant NCDs.

**Methods:**

The TROPIC project draws on the concept of ‘knowledge exchange’ between policy developers (individuals; organisations) and researchers to deliver a knowledge broking programme that maps policy environments, conducts workshops on evidence-informed policy making, supports the development of evidence-informed policy briefs, and embeds evidence-informed policy making into organisational culture. Recruitment of government and nongovernment organisational representatives will be based on potential to: develop policies relevant to obesity, reach broad audiences, and commit to resourcing staff and building a culture that supports evidence-informed policy development. Workshops will increase awareness of both obesity and policy cycles, as well as develop participants’ skills in accessing, assessing and applying relevant evidence to policy briefs. The knowledge-broking team will then support participants to: 1) develop evidence-informed policy briefs that are both commensurate with national and organisational plans and also informed by evidence from the Pacific Obesity Prevention in Communities project and elsewhere; and 2) collaborate with participating organisations to embed evidence-informed policy making structures and processes. This knowledge broking initiative will be evaluated via data from semi-structured interviews, a validated self-assessment tool, process diaries and outputs.

**Discussion:**

Public health interventions have rarely targeted evidence-informed policy making structures and processes to reduce obesity and NCDs. This study will empirically advance understanding of knowledge broking processes to extend evidence-informed policy making skills and develop a suite of national obesity-related policies that can potentially improve population health outcomes.

## Background

Policies targeting obesogenic environments and behaviours are critical to counter rising obesity rates and lifestyle-related non-communicable diseases (NCDs). Integrating evidence into appropriate and effective public policy is challenging, given that researchers, policy developers and decision makers (politicians) operate in different cultures [1-4], with different agendas, timelines and priorities that often constrain the use of evidence to inform policies [1]. Therefore, effective exchange of knowledge between evidence-producers (researchers and others) and evidence-users (those who initiate, develop, select, approve, implement and evaluate policy options) is critical to evidence-based policy development [1].

In the case of obesity-prevention, evidence-informed policies require access to evidence on the causes of obesity, as well as the implications of failing to address the immense social, health and economic costs of obesity and concomitant NCDs. However, the onus is on researchers to produce timely and relevant evidence that is readily accessible to policy developers. Policy makers need to communicate their priorities, evidence needs and timelines to evidence producers.

While some targeted obesity-prevention initiatives have reduced obesity, at least in the short term, multi-faceted efforts are needed at a population level. Evidence from rigorous intervention studies can inform policies and practices. For example, the Pacific Obesity Prevention in Communities (OPIC) project comprised community-based interventions and analytical studies (policy; sociocultural; economic) to prevent adolescent obesity in four countries (Fiji; Tonga; New Zealand; Australia) [5]. However, community-based interventions appear to be insufficient to reduce obesity at a population level, at least in Pacific nations [6,7]. The alarming escalation in the prevalence of obesity and related diseases, together with recognition of the need for a suite of policies to address obesity and political commitment to reducing obesity has created a “policy window” (Kingdon 1995) that optimises opportunities for developing policies [8] to reduce obesity.

There appears to be a policy window in the Pacific Republic of Fiji, which is governed by a military regime that has streamlined policy-making processes and is committed to improving the health of all Fiji citizens [9]. Policy windows of this nature make it even more imperative to understand the most effective approaches to obesity prevention in order to develop a suite of evidence-based policies that are likely to be effective. Policies that are developed and owned by national authorities [10] and reflect the local context [11], are fundamental to achieving public health goals.

The current TROPIC (Translational Research for Obesity Prevention in Communities) project draws on evidence from the Pacific OPIC study, and elsewhere, to heighten awareness of the high prevalence of obesity in Fiji and, importantly, inform policies that can contribute to obesity reduction, either directly or indirectly.

### Evidence-informed policy making

“Evidence-informed decision making” refers to the use of evidence to inform decisions [12,13], while “evidence-informed policy making” refers to the use of evidence to develop policies and/or advocacy statements that support policies. In this paper, the term “evidence*”* refers to academic evidence (e.g. peer-reviewed journals and books), “grey literature” (e.g. reports and health statistics), and “tacit knowledge” (e.g. past experiences; organization-specific knowledge; community contextual knowledge) [14]. The best available evidence is accessible (available; affordable; appropriate language for audience), timely and relevant (to obesity and the local context) [15]. The production of accessible, relevant evidence by evidence producers is only one of many factors that influence policy decisions [16].

The process of translating research knowledge to policy and practice has been variously framed as knowledge transfer [17,18], knowledge translation [19], knowledge exchange [20] and diffusion of innovations [21]. Each of these approaches incorporate the concept of transferring knowledge from evidence-producers to evidence-users. Evidence-informed policy making is however a much more complex undertaking than promoting research utilisation, often requiring a change in the way that business is conducted [1]. The uptake of evidence is more successful when there is a two-way information flow rather than a unidirectional push of information from evidence-producers to evidence users [22]. Knowledge *exchange* highlights the building of collaborations between evidence producers and evidence users from the outset [19], an important component given that successful relationships predict evidence-informed policy making [15,21,23]. Therefore, evidence producers (researchers) need to understand policy making processes and the culture in which policy-formulation occurs, while policy developers need a better understanding of the nature and utility of research.

Knowledge exchange also requires evidence-users (policy-developers and decision-makers) to have the commitment and skills to effectively access and critically analyse the best available evidence, and apply it to policy documents and decisions [12]. This requires building both individual capacity to access and utilise evidence and organisational processes and structures to support evidence-informed policy development culture. The use of evidence in policy making is also determined by the value that individuals and organisations place on evidence use [24]. A key factor in evidence use is the need for evidence producers and evidence users to understand the others’ goals, timelines, resources and constraints. Organisational components are stronger predictors of evidence- informed policy making than individual factors [1,13,25-27], therefore it is important that decision-making organisations to have the culture, structures and processes in place to support evidence- informed policy making activities [26]. It is clear that strategies to promote evidence-informed policy making need to address multiple factors in what is recognised as “a complex decision-making environment” [1,2].

### A knowledge broking approach

Knowledge brokers have been employed to bridge the gap between evidence producers and evidence users [23,28], assuming various roles as managers, linkage agents (raising awareness of available evidence and facilitating policy-developer networks and exchanges) [29] and/or capacity builders [22]. The primary roles of knowledge brokers have been to increase both awareness and use of the best available evidence to inform policy [30] and/or practice [22], as well as to facilitate the dissemination and, less often, the production [31] of relevant evidence. Knowledge brokers require a range of skills in order to increase evidence-informed policy making, including : 1) reviewing and interpreting relevant evidence that is aligned with policy cycles; 2) extending policy developers’ evidence-informed policy making skills and utilisation of evidence; 3) working with policy developers and organisations to develop individual and organisational cultures that value and support evidence-informed policy making; 4) facilitating strong relationships between researchers, policy developers and policy making organisations so that there is a mutual understanding of their respective goals and cultures [23,30] and relevant evidence is produced and utilised; and 5) embedding evidence-informed policy making structures and processes into policies and practices. Key attributes required of knowledge brokers are excellent communication [21,23] and motivational skills [23] and the ability to facilitate interactions between evidence-producers and evidence-users [29].

The purpose of this paper is to describe and rationalise the research design and methods used in the TROPIC project, which draws on the concept of knowledge exchange to answer the research question: “Can a ‘knowledge broker’ influence the translation of obesity prevention research findings into practice and policy?”. The specific objectives of the TROPIC project are to:

· Extend evidence-informed decision making skills in selected partner organisations

· Use a knowledge-broking approach to increase the uptake of evidence from the OPIC project and other relevant sources in the development of policy and advocacy documents

· Expand an organisational culture that supports evidence-informed policy making

## Method design

### Overview

**Figure 1:**
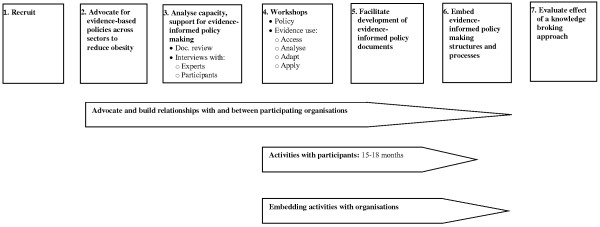
Seven phases of the TROPIC project.

The project comprises seven phases: 1) recruitment of participating organisations; 2) advocacy for more policies to address obesity; 3) analysis of evidence-informed policy making capacity and support at baseline; 4) development and delivery of tailored workshops on evidence-informed policy making; 5) support for individual participants to develop evidence-informed policy documents (policy briefs or advocacy documents) relating to obesity; 6) conduct activities to embed evidence-informed policy making into organisational culture; and 7) evaluation (see Figure 1).

The knowledge broking team will comprise a local coordinator with experience in public health and community-based obesity prevention, a research fellow and a consultant who will work on site, as well as providing remote support and advice when outside Fiji. A group of local advisors (local advisors) will be recruited on the basis of recent work with participating organisations, and their experience and expertise in policy making processes and/or governmental organisations, and/or evidence-based policy. These local advisors will advise the knowledge broking team on the organisational structure and culture of each participating organisation, especially in terms of policy making processes and the use of evidence, and suggest initiatives to sustain organisational interest in and commitment to the evidence-informed policy making process and strategies to embed evidence-informed policy making within organisations. Advice will also be provided by the TROPIC investigators as required.

#### Tailored approach

While all participating organisations will engage in a similar programme of evidence-informed policy making, the knowledge broking team will tailor the evidence-informed policy making activities to the needs of each governmental and nongovernmental organisation. Tailored programmes are desirable, given that evidence-informed policy making is context-specific [1] and that Fiji participants are likely to have a range of skills and experience in evidence-informed policy making, given the relatively small and highly mobile workforce. Each organisation will be offered a series of workshops, as well as support for the development of evidence-informed policy briefs or advocacy documents over a period of 15–18 months, the minimal duration to effect change in the utilisation of evidence-informed policy making [17,30]. Start times for each organisation’s engagement with TROPIC will be staggered to accommodate the intensive nature of the support required and the limited resources of a 2–3 person knowledge brokering team.

### The Fiji context

The Pacific Republic of Fiji is a lower-middle-income country[32] that faces a number of health challenges. Fiji has a high prevalence of obesity/overweight (in adults and children) and associated non-communicable diseases (NCDs). Many NCD-related deaths occur in the 40–59 year age group[33], thus impacting on both family and national productivity. Fiji, like many low and middle income countries, has limited economic and human resources, due in part to the relatively small population (837,271 in 2007), the small workforce charged with developing research and policy, and the high migration rates of professionals in the last three decades [34-37].

### The research context

The Pacific OPIC project yielded multiple data sets relating to adolescent obesity in Fiji, including data on the prevalence of obesity [7], obesogenic behaviors [7], sociocultural influences [38,39] and prioritisation of obesity-prevention policies relating to the food environment [40,41]. Fiji prioritised 22 policies that were recommended to the NCD taskforce [41,42]. The TROPIC project will draw on the OPIC and other relevant data sets to support participants to develop policy briefs and advocacy documents to reduce obesity in Fiji, whilst also enhancing capacity for evidence-informed policy making. The TROPIC study involves six case studies, each using similar knowledge broking strategies. Ethics approval for the study has been gained from the Fiji National Health Research Committee and the Deakin University Human Research Ethics Committee.

### Phase 1: Recruitment

Sampling will be purposive. Organisations from a range of policy sectors (e.g. health; education; agriculture) will be recruited because many obesity-related policies are developed outside the public health domain [43]. Further, non-health sectors within government often have more power to get policy topics on the agenda and formulated than do health [44]. Advocacy groups will be included in the sampling frame because professional groups and/or non-governmental organisations (NGOs) have often galvanised ministries into action [10,45]. The following criteria will be used to select organisations that will be invited to participate in TROPIC:

· Potential to make or influence policies that improve food and/or physical activity environments

· Wide demographic (ethnic group; religion; urban/rural) representation to ensure a broad reach (e.g. key ethnic and religious groups; urban and rural settings), thereby maximising the potential impact of policies

· Capacity to release and support staff to participate in KE-TROPIC activities

· Potential to share evidence-informed policy making knowledge and skills, both within and between organisations

· Previous relationships with the OPIC team, as strong relationships predict successful knowledge brokering/evidence-informed policy making [26,46].

Each participating organisation will be asked to nominate a senior staff member as a focal/contact person. For each organisation, between five and 12 staff members (TROPIC participants) who are engaged in policy development will participate in the study, as a critical mass of individuals within an organisation predicts the use of evidence-informed decision making [2,23].

### Phase 2: Advocacy for evidence-informed policies across sectors to prevent obesity

The knowledge brokering team will advocate for the development of policies that have the potential to impact on obesity at a population level, especially in non-health sectors where there is likely to be limited awareness of either interrelationships between environments, obesity and health [47,48], or of the impact of obesity on population health and national economies.

### Phase 3: Analysis of capacity and support for evidence-informed policy making

An audit of each organisation’s goals, structures and primary activities will be conducted in order to tailor the TROPIC programme to the specific needs of each organisation, thus increasing the potential for the programme to be effective [23]. National and organisational documents with relevant mission statements, goals and corporate plans will be scanned in order to determine: 1) potential priority areas for mutually advantageous policy briefs (commensurate with organisational policies, priorities and policy gaps), 2) organisational support for evidence-informed policy making, and 3) structures and processes in place for evidence-informed policy making.

In addition, semi-structured interviews (participant interviews) will be conducted with the TROPIC participants in order to determine participants’ knowledge about and experience with generating and/or using evidence, research, policy development and evidence-informed decision-making, as well as participants’ perceptions of enablers and barriers to evidence-informed policy making. A second set of semi-structured interviews (expert interviews) will be conducted with participants who have an in- depth knowledge of and recent experience with one or more of the participating organisations. The aim of these expert interviews is to determine interviewees’ perceptions of organisational culture, resources and support for evidence-informed policy making, thus providing a local ‘outsider’s’ perspective. Questions will probe: current internal and external facilitators and barriers to evidence use [1]; organisational structures that are in place to access, make sense of and utilise evidence to develop policies; and other factors that could facilitate the translation of evidence into policy.

The 2009 survey tool “*Is Research Working for You? A Self-Assessment Tool and Discussion Guide for Health Services Management and Policy Organisations”* (Is Research working for you? survey)[49] will be administered to participants prior to the workshops in order to assess evidence-informed decision making skills and perceived support for evidence-informed policy making. This survey tool was developed and revised by the Canadian Health Services Research Foundation to assist health service delivery organisations to examine strengths and weaknesses in evidence-informed decision-making [50]. The survey tool comprises 88 items and measures perceptions of culture (values, attitudes to evidence use; intentions to use evidence), and use of evidence (acquire; assess; adapt; apply) at both organisational and individual levels. The tool has demonstrated strong response variability and adequate discriminant validity [25].

### Phase 4: Workshops

A series of workshops will be provided for each organisation. It is anticipated that the workshops will address: policy and policy cycles, the Fiji policy making environment; relationships between policy and obesity; definitions of evidence; evidence sources; skills in accessing, analyzing, synthesising and adopting evidence for policy documents; policy/advocacy topic areas that could potentially impact on obesity and are commensurate with national/organisational goals (see Figure 1). The organisational audit data collected prior to the workshops will inform the depth and time allocated for each topic area. Workshops will combine information delivery and interactive practical sessions to reinforce learning.

### Phase 5: Facilitated development of evidence-informed policy documents

The knowledge brokering team will support participants individually and/or in small groups to develop evidence-informed policy briefs or advocacy statements on mutually agreed topics in order to: 1) reinforce skills gained during workshops, and 2) ensure that there is relevant evidence available to inform the brief. As with the workshops, the nature and the level of the support will be informed by the preliminary analyses of individual and organisational strengths and requirements. Support may also include the production of written guidelines on evidence-informed policy making processes and summaries of relevant evidence.

### Phase 6: Embedding of evidence-informed policy making within participating organisations

Embedding activities will include the: 1) strengthening of relationships between evidence-producers and evidence-users, 2) building of a critical mass of people within and between organisations who have the skills to acquire, assess and adapt evidence to inform policy, 3) advocacy for clear structures and processes to support evidence-informed decision making, as well as formal recognition and rewards for evidence-informed policy making, and 4) facilitation of networks of evidence-informed policymakers within and between organisations.

### Phase 7: Evaluation of the effectiveness of a knowledge brokering approach

The effectiveness of a knowledge broker in increasing the use of evidence in the development of policy documents to reduce obesity will be identified through examination of several data sets (see Table 1), thus allowing more rigorous and detailed evaluation.

**Table 1:** Measures to determine effectiveness of the TROPIC knowledge-broking process on evidence-informed policies to reduce obesity

**Measure**	**Purpose**	**Sample**	**Time applied**	**Analysis**
Semi- structured interviews	Identify understanding of and experience with evidence, policy and evidence-informed policy making	All individual participants	Pre-TROPIC	· Descriptive
				· Thematic
Is Research Working for You? survey (CHSRF)	Identify perceived evidence-informed decision making skills and resources at organisational and individual levels	All individual participants	Pre-TROPIC	
Semi-structured interviews	Identify perceptions of organisational culture and resources to support evidence-informed policy making	Purposive sampling of one expert per org.	Pre-TROPIC	· Descriptive
				· Thematic
Process diary completed by TROPIC team	Identify resources utilised (time spent; personnel; purpose) in knowledge broking activities at individual and organisational levels		Ongoing	· Descriptive statistics for level of individual participation and TROPIC outputs
Is Research Working for You? Survey (CHSRF)	Identify perceived changes in EIDM skills and resource at organisational and individual levels following TROPIC	All participants	Post-TROPIC	· Repeated measure ANOVAs
Interviews	Identify perceived impact of TROPIC on individuals’ roles, organisational position and future careers	All participants	Post-TROPIC	· Descriptive
· Structured (electronic)				· Thematic
· Semi- structured (face-to- face)				
Structured interviews	Identify perceived impact of TROPIC on organisation	High-level officer from each organisation (n = 6)	Post-TROPIC	· Descriptive
				· Thematic

#### Evidence-informed decision-making

Participants will complete the survey tool “*Is Research Working for You? A Self-Assessment Tool and Discussion Guide for Health Services Management and Policy Organizations” (Is Research Workingfor you? survey)* prior to and following the completion of the TROPIC programme. This will facilitate assessment of the changes in the valuing and utilisation of evidence-informed decision-making.

#### Perceptions of the TROPIC programme

Individual and organisational perceptions of the effectiveness of the TROPIC programme will be elicited by interviews that will complement the survey data by examining the impact of TROPIC on individuals and organisations. Interviews will be conducted with all participants in the TROPIC programme, and a high-level officer (non-participant) from each participating organisation (see Table 1).

#### Participant interviews

Participant interviews will comprise two components. First, participants will complete a set of structured questions that will be emailed to them at the end of Phase 6. Questions will address the skills that participants may have gained from the TROPIC programme, career opportunities that have arisen consequent to TROPIC and areas for improvement, and enablers and barriers to the development of policy documents. Second, individuals’ responses from pre- and post-interviews and surveys will be examined in order to develop a set of interview questions specific to each individual. A 10–15 minute face-to-face interview will then be conducted with each participant to seek further detail, for example, explanations for a response shift (unexpected/negative changes in the direction of responses [51]).

### Process evaluation

The knowledge brokering team will keep a diary of all evidence-informed decision making activities initiated either by the TROPIC team, or the participants and/or their organisations. Based on Dobbins et al. (2009), the diary will detail all TROPIC activities within and across organisations, including details about who initiates and responds to an activity, the nature of the request, type of response and resources involved in this engagement (e.g. personnel; time). The team will also maintain a diary of the outputs arising from TROPIC, specifically the completion and progression of policy briefs and advocacy documents, as well as actions related to embedding of evidence-informed decision-making practice (e.g. development of policy units, key performance indicators).

### Analysis

The various data sets (interviews; surveys; process) will be analysed in order to examine the effect of the TROPIC knowledge-broking approach on the evidence-informed policy making of individual participants, as well as participating organisations. Participant interviews will be transcribed, checked and uploaded onto NV8 (QSR, Melbourne), software for the analysis of qualitative data. Data will be coded using a combination of pre-determined codes relating to the research questions, as well as codes that arise from the transcripts. Data will then analysed at several levels: descriptive categories; underlying themes; and constant comparative analysis in order to compare results from different sub-groups (e.g. the level of engagement during TROPIC). Analyses of interviews will be collaborative, with the first and second authors conducting independent analyses and then comparing and reconciling any differences. Collaborative analysis is critical when undertaking exploratory studies, and particularly in the case of TROPIC data where analyses require careful contextual interpretation. Survey data will be entered into SPSS (V19). Descriptive statistics will be used to summarise scores on the various dimensions of the evaluation tool. Changes in pre- and post-TROPIC scores across the different organisational and individual dimensions will be assessed using repeated measures ANOVAs. Process data will be analysed descriptively to determine relationships between resource-use and level of engagement in TROPIC activities (e.g. workshop completion), and outputs (e.g. policy briefs; structures to support evidence-informed policy making).

## Discussion

The TROPIC project will contribute significantly to knowledge about the effectiveness of a knowledge brokering approach to increase the utilisation of obesity-related evidence in policy and advocacy documents by employing a mixed-methods approach to analysis and evaluation. The combination of qualitative and quantitative data sets is important, given the exploratory nature of the project and the need to describe and explain cultural and behavioural factors influencing evidence-informed decision making. The mixed-method analyses (interviews; surveys) will enable in-depth examination of relationships between evidence-informed policy making activities and outputs in relation to the level of engagement with TROPIC activities, as well as how these might be moderated by intrapersonal variables (e.g. education background). Further, the use of a repeated measures design will enable important information about more subtle changes in evidence-informed decision making to be discerned.

The emphasis on process evaluation is based on the concept that knowledge translation is a process rather than a single event [2,52]. The TROPIC project will examine the processes involved in enhancing evidence-informed policy making and will analyse relationships between level of engagement, resources used and evidence-informed policy making outputs.

While the duration of the TROPIC initiatives is relatively short, positive changes in evidence-informed policy making are predicted, given that the project will: 1) reflect national and organisational goals of participating organisations, 2) provide intensive support for participants to develop policy documents, and 3) build on strong researcher-end-user relationships established in the five-year OPIC study. The use of a knowledge brokering team rather than an individual knowledge broker is advantageous, given the complexity of integrating evidence-informed policy making into any policy environment [1] and the number of roles required of a knowledge broker [53].

Public health interventions have rarely targeted evidence-informed policy making structures and processes synergistically to reduce obesity and NCDs. This study will advance our understanding of knowledge-broker approaches to extend evidence-informed policy making skills in relation to obesity- prevention policies. However, the evidence-informed policy making skills gained during TROPIC are generic and can be transferred to any policy area; the transferability of skills is especially important in a LMIC with limited policy making resources.

Evaluation of the knowledge brokering *processes* in the TROPIC project design are especially important in countries like Fiji, given the additional challenges for evidence-informed policy development in LMICs. Specifically, LMICs often have fewer resources and less capacity to either access or adapt evidence for policy documents [10], or foster a culture that supports and extends evidence-informed policy making. These limitations make it difficult to foster and sustain a culture, structures and processes that support evidence-informed policy making. Many LMICs have the additional challenge of multiple competing health priorities [10]. Ironically, the need for evidence-informed policies to reduce obesity and NCDs is greater in LMIC countries than in many western countries; more than 50% of deaths in LMIC countries are NCD-related and 30% of these deaths occur before the age of 60 [10]. The TROPIC project will make a significant contribution to the evaluation and understanding of building evidence-informed policy making capacity, not only in LMICs, but also in more wealthy countries that have a high prevalence of obesity.

## Competing interests

The authors declare that they have no competing interests.

## Authors’ contributions

HM reviewed the literature, led the design process and drafted the manuscript. GW contributed to the study design, in particular providing advice on the applicability of the study to the local setting. MM contributed to the study design and critically reviewed the manuscript. PK contributed to the study design and critically reviewed the manuscript. MMcC contributed to the study design and critically reviewed the manuscript. WS critically reviewed the manuscript. BS contributed to the study design and critically reviewed the manuscript. All authors read and approved the final manuscript.

## Pre-publication history

The pre-publication history for this paper can be accessed here:

http://www.biomedcentral.com/1471-2458/12/552/prepub
